# Expression of Angiotensin II Receptor-1 in Human Articular Chondrocytes

**DOI:** 10.1155/2012/648537

**Published:** 2012-12-30

**Authors:** Yuki Kawakami, Kosuke Matsuo, Minako Murata, Kazuo Yudoh, Hiroshi Nakamura, Hiroyuki Shimizu, Moroe Beppu, Yutaka Inaba, Tomoyuki Saito, Tomohiro Kato, Kayo Masuko

**Affiliations:** ^1^Department of Biochemistry, Institute of Medical Science, St. Marianna University School of Medicine, Kawasaki-shi, Kanagawa 2168511, Japan; ^2^Department of Orthopaedic Surgery, School of Medicine, Yokohama City University, Yokohama-shi, Kanagawa 2360004, Japan; ^3^Department of Frontier Medicine, Institute of Medical Science, St. Marianna University School of Medicine, Kawasaki-shi, Kanagawa 2168512, Japan; ^4^Department of Joint Disease and Rheumatism, Nippon Medical School, Bunkyo-ku, Tokyo 1138603, Japan; ^5^Department of Orthopaedic Surgery, St. Marianna University School of Medicine, Kawasaki-shi, Kanagawa 2168511, Japan; ^6^Graduate School of Nutritional Science, Sagami Women's University, Sagamihara-shi, Kanagawa 2520383, Japan

## Abstract

*Background*. Besides its involvement in the cardiovascular system, the renin-angiotensin-aldosterone (RAS) system has also been suggested to play an important role in inflammation. To explore the role of this system in cartilage damage in arthritis, we investigated the expression of angiotensin II receptors in chondrocytes. *Methods*. Articular cartilage was obtained from patients with osteoarthritis, rheumatoid arthritis, and traumatic fractures who were undergoing arthroplasty. Chondrocytes were isolated and cultured in vitro with or without interleukin (IL-1). The expression of angiotensin II receptor types 1 (AT1R) and 2 (AT2R) mRNA by the chondrocytes was analyzed using reverse transcription-polymerase chain reaction (RT-PCR). AT1R expression in cartilage tissue was analyzed using immunohistochemistry. The effect of IL-1 on AT1R/AT2R expression in the chondrocytes was analyzed by quantitative PCR and flow cytometry. *Results*. Chondrocytes from all patient types expressed AT1R/AT2R mRNA, though considerable variation was found between samples. Immunohistochemical analysis confirmed AT1R expression at the protein level. Stimulation with IL-1 enhanced the expression of AT1R/AT2R mRNA in OA and RA chondrocytes. *Conclusions*. Human articular chondrocytes, at least partially, express angiotensin II receptors, and IL-1 stimulation induced AT1R/AT2R mRNA expression significantly.

## 1. Introduction

The renin-angiotensin-aldosterone system (RAS) is an essential regulator of the fluid and electrolyte balance in the human body, and it thereby controls blood pressure. The RAS system has also been implicated in atherosclerosis and cardiac hypertrophy, in which it is considered to induce cardiovascular system remodeling (reviewed in [1]). 

Renin is secreted by the mesangial cells of the kidney, and it triggers the release of angiotensin I (Ang I) from the N terminus of circulating angiotensinogen (the substrate of renin). Angiotensin-converting enzyme (ACE) then cleaves angiotensin II (Ang II) from the release molecule. Ang II comprises 8 amino acids of the N terminus of Ang I [2, 3]. 

Ang II functions by binding to specific Ang II receptors [2, 4]. The Ang II receptors identified thus far are AT1 receptor (AT1R) and AT2 receptor (AT2R), with distinct tissue distributions [5, 6]. AT1R is responsible for the major functions of Ang II, whereas AT2R is suggested to act as a counter-regulatory receptor [4]. Thus, specific inhibitors of AT1R (Ang II receptor blockers; ARBs) have been widely applied in clinical strategies against hypertension. 

The activities of the RAS system are not limited to the cardiovascular system. “Local RAS systems” have been found to be present in multiple tissues and organs (reviewed in [3]). For example, there is accumulating evidence to strongly suggest that the RAS system might be actively involved in the pathophysiology of arthritis, including rheumatoid arthritis (RA) and osteoarthritis (OA) [7–10]. Further, it was reported that the synovial joint contains renin and ACE, and the ratio of the amount of active renin in synovial fluid to that in plasma was significantly high, suggesting the local conversion of inactive renin to the active form [7, 8]. 

In RA, human fibroblast-like synoviocytes were shown to express AT1R, and Ang II was found to protect synoviocytes from apoptosis, playing a role in synovial expansion in vitro [11]. Furthermore, blocking of the RAS system by ARB improved RA in animal models [9, 10]. The antiarthritic effect of ARB might be attributable to the suppression of the antigen-specific immune response by ARB, as reported by Sagawa et al. [9], and Iwamoto et al. also speculated that ARB can suppress the expression of monocyte chemoattractant protein (MCP)-1 in rheumatoid synoviocytes [12].

Despite these findings, the precise role of the RAS system in bone and cartilage is not understood. Articular chondrocytes are the only cell type composing articular cartilage in joint tissue. Although chondrocytes are in the avascular environment, they are effected by molecules in the synovial fluid which penetrate the extracellular matrix of cartilage, as well as autocrine/paracrine molecules. If an adequate amount of Ang II is present in the joints, the chondrocytes might respond to this Ang II stimulus since they express angiotensin receptors. It is unknown whether the chondrocytes of arthritic patients express AT1R and/or AT2R.

In this study, we investigated the expression of the angiotensin II receptors in articular chondrocytes from arthritic patients in order to clarify the role of RAS in arthritis.

## 2. Materials and Methods

### 2.1. Cells

Human articular chondrocytes were obtained from patients with OA (*n* = 14; from 12 knee and 2 hip joints), RA (*n* = 14; from 14 knee joints), and traumatic fractures (*n* = 13; from 13 hip joints), who underwent joint arthroplasty at St. Marianna University School of Medicine Hospital or Nippon Medical School Hospital. All patients were examined by a certified rheumatologist or orthopedic surgeon and diagnosed on the basis of the criteria developed by the American College of Rheumatology (http://www.rheumatology.org/practice/qmc/criteria_old.asp). The fracture patients had no history of joint disease and thus served as a control. Written informed consent was obtained from all patients, and the protocol was approved by the institution's ethics committee. The study was performed in compliance with the Declaration of Helsinki proposed by the World Medical Association in 1964.

Chondrocytes were obtained as reported previously [2]. In brief, after the synovial tissue was carefully removed, the cartilage was minced, washed, and treated with 0.5% (w/v) collagenase at 37°C for 5 hours. Isolated chondrocytes were then washed and culturedin vitro as a monolayer in Dulbecco's modified Eagle's medium (DMEM) supplemented with 10% fetal calf serum (FCS) and antibiotics. The FCS used in the study was inactivated by incubation at 56°C for 30 min. The attached cells (P0) were grown on type I collagen-coated culture dishes, and cells at subconfluence (P1) were used in the experiments. The phenotypes of the differentiated cells used in the experiments were confirmed by macroscopic observation and on the basis of the expression of type II collagen and aggrecan mRNA (data not shown).

### 2.2. Reagents

Rabbit polyclonal anti-human AT1R antibodies (Abs) were purchased from Assay Designs (Ann Arbor, MI, USA; for immunohistochemistry) and from Alomone Labs, Ltd. (Jerusalem, Israel; for western blotting and flow cytometry). Antiglyceraldehyde-3-phosphate dehydrogenase (GAPDH) was purchased from Abcam Ltd. (Cambridge, UK).

#### 2.2.1. In Vitro Stimulation of Chondrocytes

Chondrocytes were serum starved in DMEM containing 0.5% FCS for 24 h prior to the experiments, and they were then stimulated with 1–10 ng/mL recombinant human interleukin-1*β*(IL-1) (R&D Systems; Minneapolis, MN, USA) for 24 h. Cells not treated with IL-1 were used as the controls. Cell viability was not affected by IL-1 during the culture period, as confirmed by a trypan blue exclusion test (data not shown). The stimulated chondrocytes and culture supernatants were collected and used in the subsequent analyses. 

### 2.3. Reverse Transcription-Polymerase Chain Reaction (RT-PCR)

mRNA was extracted from the cultured cells and converted to cDNA as described previously [13]. The primer sequences were as published previously [14], and the oligonucleotides used were those synthesized by Bex Co. Ltd. (Tokyo, Japan). 

PCR was performed with the following conditions: 94°C for 2 min; 35 cycles (for GAPDH and AT1R) or 45 cycles (for AT2R) of 94°C for 30 s, 58°C for 1 min, and 72°C for 1 min, and extension at 72°C for 10 min.

The amplified products were visualized using 2% agarose gel electrophoresis.

### 2.4. Quantitative PCR

Total RNA obtained from the chondrocytes was converted into cDNA and purified using a PCR purification kit (Qiagen; Hilden, Germany). Quantitative PCR was performed using specific primers and ABI Prism 7000 (Applied Biosystems, Foster City, CA, USA), according to the manufacturer's protocol. The primer sequences for AT1R (GenBank no. NM_000685) and AT2R (GenBank no. NM_000686) were as follows. AT1R forward primer, 5′-CAGATGACGGCTGCTCGAAG-3′ and reverse primer, 5′-TGGAAACTGGACAGAACAATCTGG-3′ and AT2R forward primer, 5′-GTGTGTTTAGGCACTAAGCAAGCTG-3′ and reverse primer, 5′-GTTCACAAGCCCGAAGTGAAGA-3′ were used.

The oligonucleotides were synthesized by Takara Bio Co. Ltd (Otsu, Japan). 

### 2.5. Western Blotting

Whole cell lysates were extracted from the cultured cells by using standard lysis buffer (20 mM Tris-HCl, 250 mM NaCl, 1% NP-40, 1 mM dithiothreitol, 10 mM NaF, 2 mM Na_3_VO_4_, 10 mM Na_4_P_2_O_7_, and protease inhibitor cocktail) (Roche; Mannheim, Germany) and stored at −30°C until use. The protein concentration was determined using the Bradford method (Bio-Rad protein assay reagent; BioRad Laboratories; Hercules, CA, USA). The lysates were mixed with the dye used to assess migration and subjected to sodium-dodecyl-sulfate (SDS-) polyacrylamide gel electrophoresis (PAGE). After transfer to polyvinylidene difluoride membranes, the primary Ab was added. The working concentrations of the primary Abs were as follows: 1 : 1,000 for anti-AT1R Ab and 1 : 10,000 for anti-GAPDH Ab. The secondary Abs anti-rabbit Ig-HRP and anti-mouse secondary Ig-HRP were used at 1 : 5,000 and 1 : 10,000, respectively. Abs were diluted with 1% bovine serum albumin (BSA)/phosphate buffered saline (PBS)/0.1% tween 20. The membranes were then washed with PBS/0.1% tween 20, and the signals were visualized using an extended cavity laser (ECL) system (GE Healthcare Bio-sciences KK; Tokyo, Japan). 

### 2.6. Immunohistochemistry

Immunohistochemical analysis was performed as reported previously [2]. Briefly, articular cartilage tissue specimens were fixed in 4% paraformaldehyde/PBS. After decalcification, the sections were embedded in paraffin and cut into 4 *μ*m thick sections. After deparaffinization, sections were incubated with proteinase K (DAKO, Glostrup, Denmark) and Block Ace (Dainippon Pharmaceuticals, Osaka, Japan), followed by incubation with the primary Ab (1 : 1000 anti-AT1R), and detected with the secondary Ab and LSAB + System-HRP (DakoCytomation; Glostrup, Denmark). As a negative control, 1 *μ*g/mL normal rabbit IgG was used instead of the primary Ab.

### 2.7. Flow Cytometry

Cells were incubated with or without 10 ng/mL IL-1 for 24 h and then collected using 5 mM EDTA. They were washed, blocked with 10% Block-Ace (DS Pharma Biomedical, Suita, Japan) in PBS, and incubated for 1 h with 5 *μ*g/mL anti-human AT1R Ab or 5 *μ*g/mL rabbit immunoglobulin as the isotype control. After the cells were washed twice, they were incubated with goat anti-rabbit fluorescein isothiocyanate (FITC) conjugate for 1 h in the dark. The cells were washed and immediately analyzed using a FACS Calibur system (Nippon Becton Dickinson; Tokyo, Japan), and calculations were performed with CellQuest analysis software (Nippon Becton Dickinson).

### 2.8. Statistical Analyses

Statistical analyses were performed using Prism software (GraphPad Software Inc.; San Diego, CA, USA). The results are represented as the mean ± SD. Student's *t* test was used for comparisons between groups. A *P* value <0.05 was considered significant.

## 3. Results

Human articular chondrocytes express angiotensin receptors

**Figure 1:**
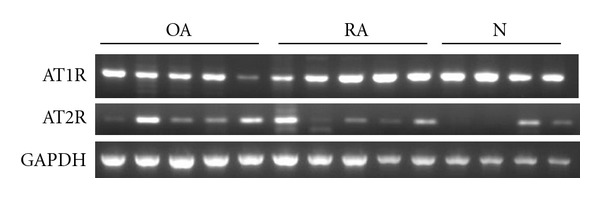
Expression of angiotensin receptors in human chondrocytes. Representative results of RT-PCR using 5 OA, 5 RA, and 4 fracture (control) samples are shown. AT1R: 255 bp, AT2R: 191 bp, GAPDH: 598 bp.

We used RT-PCR to investigate the expression of angiotensin receptor mRNA by chondrocytes. The results are shown in Figure 1. As demonstrated, both AT1R and AT2R were expressed in all samples tested, although there was considerable variation in the expression levels among the samples.

**Figure 2:**
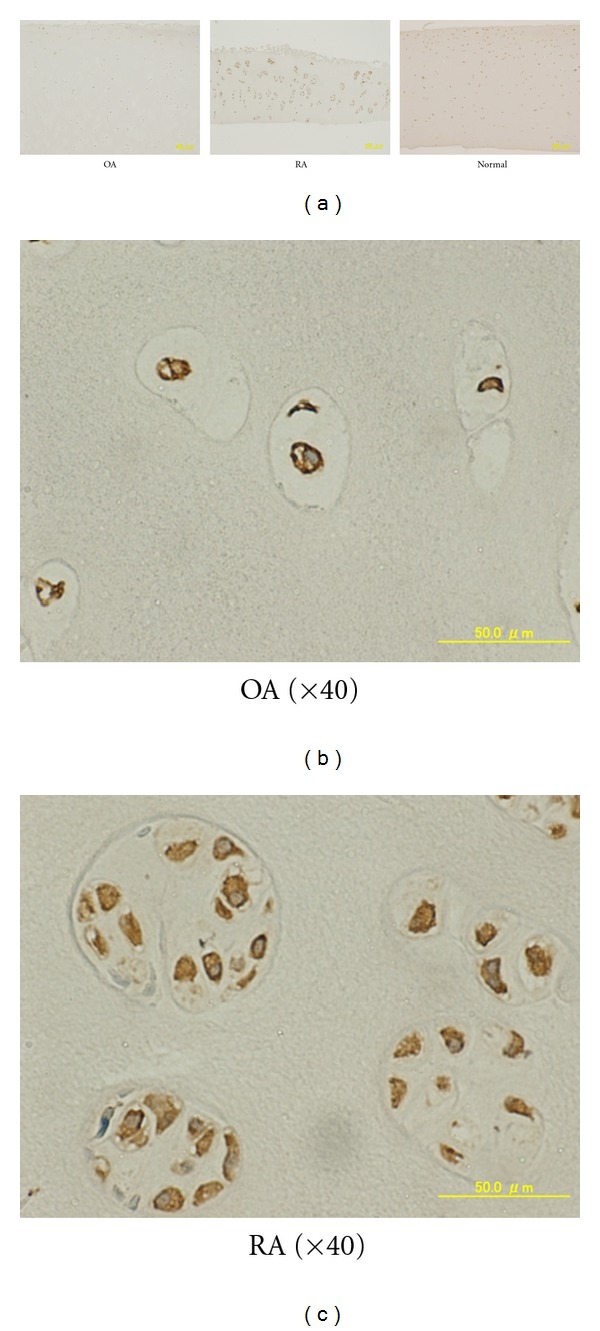
Immunohistochemical analysis of AT1R expression in human cartilage. Representative results are shown. (a) Original magnification: ×4. (b) An OA sample (×40). (c) An RA sample (×40).

Since AT1R is the dominant Ang II receptor, we further confirmed the expression of this receptor at the protein level. The chondrocytes in the cartilage obtained from OA, RA, and fracture patients were stained positively for AT1R (Figures 2(a), 2(b), and 2(c)). Despite some degree of degeneration of cartilage, such as fibrillation in OA and cluster formation of chondrocytes in RA, no significant pattern was apparent in the distribution of AT1R-expressing chondrocytes within the cartilage. AT1R was strongly expressed on cell surface and showed moderate expression in plasma.

### 3.1. IL-1 Upregulates AT1R mRNA but not AT1R Protein in Chondrocytes

To clarify the role of inflammatory mediators in AT1R expression, we stimulated chondrocytes with a representative proinflammatory cytokine, IL-1, and analyzed them for receptor expression.

**Figure 3:**
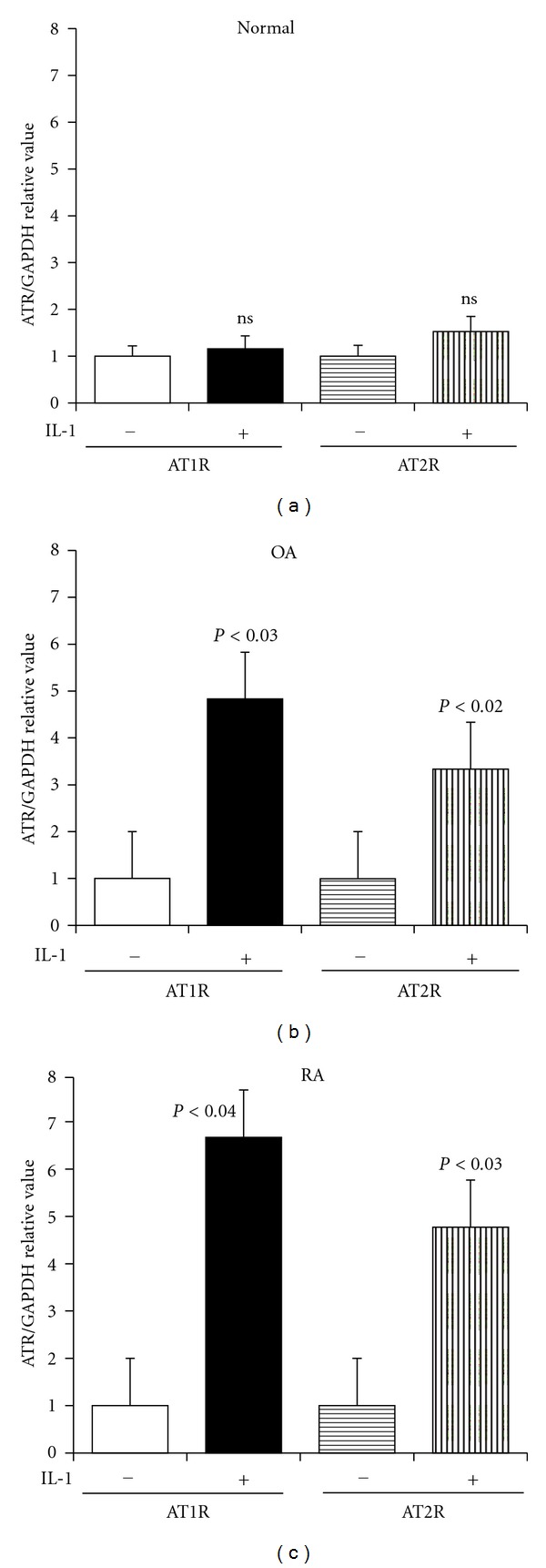
IL-1-induced upregulation of AT1R/AT2R mRNA expression in chondrocytes. Results of the quantitative PCR analyses are shown. (a) Normal (*n* = 3) (b) OA (*n* = 4) (c) RA (*n* = 4).

As shown in Figure 3, levels of both AT1R and AT2R mRNA were similar between groups. However, after IL-1 stimulation, chondrocytes tended to exhibit higher expression levels of both AT1R and AT2R mRNA than nonstimulated ones. The difference of both AT1R and AT2R mRNA level was significant in OA and RA groups. (Figure 3(b)).

**Figure 4:**
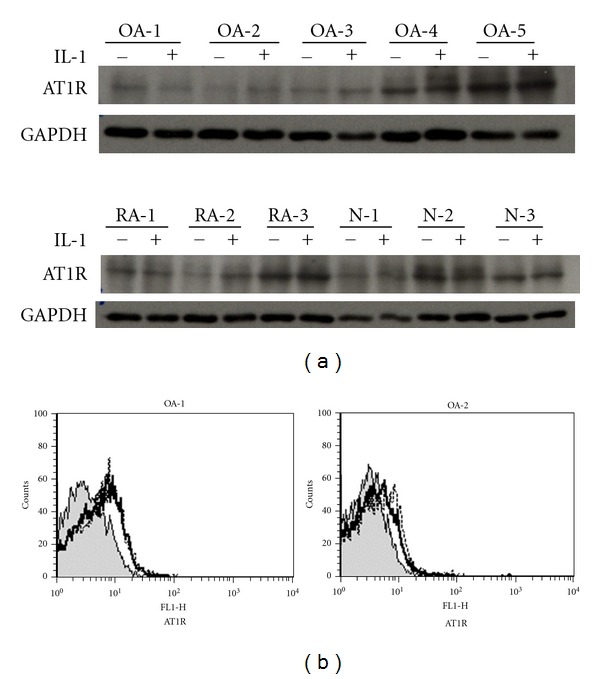
AT1R protein expression in chondrocytes. (a) *Western Blotting*. Chondrocytes with or without IL-1 stimulation were analyzed: AT1R: approximately 60 kD (see text), GAPDH: 36 kD. (b) *Flow Cytometry*. A representative result is shown, shaded area: control staining, solid line: nonstimulated chondrocytes, dotted line: IL-1-stimulated chondrocytes. Representative results of 2 OA samples are shown.

Next, we investigated whether the IL-1 upregulates AT1R expression at the protein level. The western blot analysis, however, showed that the difference in AT1R protein expression between the IL-1-stimulated and nonstimulated chondrocytes was not significant in most samples, although some samples showed that it was slightly greater in the stimulated cells (Figure 4(a)). Interestingly, AT1R was detected as one band at approximately 60 kD using 2 different AT1R-specific Abs despite of a calculated molecular weight of 41 kD (data not shown), suggesting a posttranslational modification in chondrocytes in all groups, as reported previously [15, 16]. We further tested the cell surface expression of AT1R by using flow cytometry. The results confirmed the cell surface expression of AT1R in a subset of OA chondrocytes (Figure 4(b)), but the level of AT1R expressed did not change with IL-1 stimulation. 

## 4. Discussion

In this study, we examined the expression of angiotensin receptors in chondrocytes and the upregulation, at least in part, of AT1R mRNA production or mRNA stability in chondrocytes by IL-1 stimulation. To our knowledge, this is the first report to show AT1R expression in human articular chondrocytes. 

Our results prove that not only synoviocytes but also articular chondrocytes express AT1R. The function of the RAS system in cartilage and bone remains unclear and is being investigated in our laboratory, but considering the known effects of Ang II, the RAS system may be implicated in the expression of matrix metalloproteinases (MMPs) and tissue remodeling in cartilage matrix, as has been reported in the case of other tissues, such as the heart [17–19]. Specifically, Ang II is reported to be a profibrotic factor that regulates the expression of type I collagen and MMPs such as MMP-2, probably leading to the development of tissue fibrosis in the heart or other organs. Therefore, it would be of particular interest to explore whether AT1R delivers the Ang II stimulus to chondrocytes, thereby modulating the expression of cartilage matrix components. Paradis et al. reported that mice overexpressing human AT1R showed cardiac hypertrophy and remodeling with increased collagen deposition, without any changes in systolic blood pressure [17]; thus, AT1R itself, and not Ang II, might be responsible for the regulation of collagen turnover. In addition, since recent investigations suggest that Ang II might stimulate the proliferation and senescence of vascular endothelial cells [20, 21], the potential effect of Ang II in chondrocyte senescence or survival might be considered. 

We observed that when stimulated with IL-1, the chondrocytes upregulated the expression of angiotensin receptor mRNA; this was especially apparent in the OA chondrocytes. This finding may be consistent with that of a report which demonstrated the IL-1-induced upregulation of AT1R in cardiac fibroblasts [22]. In contrast, the expression of the AT1R protein in the chondrocytes (in the cell extract or on the cell surface) was not significantly enhanced by IL-1 (Figure 4). The reason for this discrepancy is not clear, but it could be that (1) the degree of IL-1R expression and/or the kinetic response to IL-1 varies in each sample; (2) that IL-1 regulates the transcription of AT1R, whereas the upregulation of translation requires another signals; (3) that only a subset of cells among the cultured chondrocytes tested express AT1R, because of which the upregulation of AT1R was not detectable in the present experiments. Using rat cardiac fibroblasts, Cowling et al. [23] observed that IL-1 increased the AT1R levels mainly by stimulating transcription and that the degree of upregulation by IL-1 differed among AT1R splice variants. We too speculated that IL-1 might alter the posttranscriptional splicing of AT1R. Thus, AT1R protein expression should be further investigated using different experimental conditions and anti-AT1R Abs with epitope specificities. 

We did not observe any significant difference in the basal level of AT1R in the OA, RA, and control chondrocytes (Figures 1 and 2). However, the expression varied considerably among samples regardless of the disease, and it has been shown that mechanical stress activates AT1R without the involvement of Ang II [24–26]. Thus, it might be possible that mechanical compression or local inflammation might transiently or locally modulate the cell surface and/or mRNA expression of AT1R in chondrocytes. The mechanism by which AT1R and AT2R expression is regulated should be analyzed in more detail in the future. 

In conclusion, we demonstrated the expression of angiotensin receptors in human articular chondrocytes. Understanding the involvement of AT1R-mediated signaling in arthritis may open up a new avenue for establishing novel antidegenerative strategies focusing on the RAS system in articular joints.
